# Supplementary material for: Correlates of long-acting reversible contraceptive (LARC) use among young women in Southern Africa: a secondary analysis from HPTN 082
Source: medRxiv. 2025 Sep 18:2025.09.16.25335943. Preprint. [Version 1] doi: 10.1101/2025.09.16.25335943 (PMC12458495; doi:10.1101/2025.09.16.25335943)
Supplement: Supplement 1 [file NIHPP2025.09.16.25335943v1-supplement-1.pdf]

Supporting Information

S1 File. Supplementary tables.

This file contains additional materials supporting the main findings:

Table 1. Demographics and baseline characteristics by LARC use.

Table 2. Baseline factors by LARC usage through Week 39.

S2 File. HPTN 082 Study Protocol.

This file contains the most recently approved HPTN 082 study protocol
